# Aberrant Methylation Inactivates Transforming Growth Factor *β* Receptor I in Head and Neck Squamous Cell Carcinoma

**DOI:** 10.1155/2009/848695

**Published:** 2009-06-14

**Authors:** Teresita Muñoz-Antonia, Mariclara Torrellas-Ruiz, Jonathan Clavell, Linda A. Mathews, Carlos A. Muro-Cacho, Adriana Báez

**Affiliations:** ^1^Department of Interdisciplinary Oncology, Moffitt Cancer Center, Tampa, FL 33612, USA; ^2^Department of Pathology, LSU Health Sciences Center, New Orleans, LA 70112, USA; ^3^Department of Pharmacology, University of Puerto Rico School of Medicine, San Juan, PR 00936, USA; ^4^Pathology and Laboratory Medicine, James A. Haley Veterans' Affairs Medical Center, Tampa, FL 33612, USA; ^5^Department of Otolaryngology, University of Puerto Rico School of Medicine, San Juan, PR 00936, USA

## Abstract

*Background*. Alterations in TGF-*β* signaling are common in head and neck cancer (HNSCC). Mutations in TGF-*β* type II receptor (*T* *β* *R-II*) occur frequently in HNSCC while TGF-*β* type I receptor (*T* *β* *R-I*) mutations are rare, suggesting that other molecular alterations in the TGF-*β* pathway are likely. To identify abnormalities in *T* *β* *R-I* expression we analyzed 50 HNSCCs and correlated the results with clinical-pathologic features. *Methods*. Hypermethylation of *T* *β* *R-I* was evaluated via methylation-specific PCR (MSP) and restriction enzyme-mediated PCR (MSRE). Mutations in exons 1 and 7, mRNA and protein expression were analyzed by direct sequencing, semiquantitative RT—PCR and immunohistochemistry, respectively. *Results. T* *β* *R-I* expression was lost in 83% HNSCCs and was linked to DNA hypermethylation of the CpG-rich promoter region in 62% of the tumors. The variants 9A/6A and Int7G24A were found in two patients. *Conclusions*. This study shows that suppression of *T* *β* *R-I* expression in HNSCC is associated with DNA hypermethylation.

## 1. Introduction

 Over 90 percent of head and neck cancers are squamous cell carcinomas (HNSCCs) that arise from the mucosal lining of the upper aerodigestive tract [1]. HNSCC is the fifth most common malignancy worldwide, with more than 500 000 new cases diagnosed each year. It is estimated that these tumors accounted for 45 700 new cases and 11 210 deaths in 2007 in the United States [2].

Patients often present with advanced stage disease and, despite combined therapy, the 5-year survival rate of approximately 50% has improved only marginally in recent years. Tumors are typically staged by combining clinical and pathological parameters of the primary tumor and its metastases [3]. There are no reliable markers of early detection and prognosis, and the overall genetic and molecular basis of HNSCC remains ill-defined. The major risk factor is epithelial exposure to tobacco and alcohol but, more recently, human papillomavirus (HPV), an etiological agent in cervical cancer, has been linked to HNSCC, especially in the oropharynx [4, 5].

HNSCCs are frequently resistant to the growth inhibition mediated by transforming growth factor-*β* (TGF-*β*). In the majority of cases, defects in the TGF-*β* type II receptor (*T* *β* *R*-*I* *I*) have been shown to play an important role in this resistance. In a subset of tumors, however, the mechanism responsible is not yet fully understood [6, 7].

The TGF-*β* superfamily is a set of multifunctional cytokines that regulate numerous cellular functions including proliferation, differentiation, organ development, wound healing, and immunity [8]. TGF-*β* effects are mediated by a membrane-bound serine/threonine kinase receptor complex, consisting of type I and type II receptors [8, 9] and their downstream signal transducers, the Smad proteins [10]. Tumor cells escape TGF-*β*-mediated growth regulation via the loss of one or more functional TGF-*β* receptors and/or Smad proteins [10]. Since these abnormalities can result in unregulated cell growth, various components of the TGF-*β* signaling pathway are considered tumor suppressor genes [9].

Genetic alterations and alterations of epigenetic information are associated with malignant transformation and progression in most cancers [11]. Modification of DNA methylation patterns and chromatin remodeling contribute to epigenetic alterations of gene expression [11]. It has been suggested that methylation silencing is as important as loss of heterogeneity or mutations in cancer development [11] and that each tumor appears to have a characteristic profile of methylated genes [12]. Mutations in *T* *β* *R*-*I* *I* have been frequently found in colon [13] and gastric [14] cancers but are less frequent in HNSCCs [15, 16] and cancers of prostate [17] and breast [18]. Mutations in *T* *β* *R*-*I* are less frequent and have been reported in lymphoma [19] and in ovarian [20, 21] and pancreatic [22] cancers. A germline mutation, *Int7G24A*, associated with susceptibility to cancer, has been detected in carcinomas of the lung [23], kidney and bladder [24], and breast [25]. One study found no somatic mutations in the *T* *β* *R*-*I* gene in 30 primary HNSCCs [26] while another found them in 4 of 21 metastatic HNSCCs [27]. Inactivating mutations of the *Smad2* gene have been detected in a small group of human colorectal, lung, hepatocellular, and cervical cancers [28]. Moreover, *Smad4/DPC4* is inactivated by somatic mutations in pancreatic, colonic, and pulmonary carcinomas [29]. While methylation of the *T* *β* *R*-*I* *I* promoter region has been reported in esophageal [30] and nonsmall cell pulmonary carcinomas [31], aberrant methylation of *T* *β* *R*-*I* has been reported both in gastric cancer cell lines and in primary gastric adenocarcinomas [32, 33]. 

Recently, we reported that Smad4 expression is significantly reduced in HPV16-positive compared to HPV16-negative HNSCCs [34]. In the same study, we detected a significant reduction in the expression of *T* *β* *R*-*I* *i*n most of the HNSCCs tested. In order to understand the molecular mechanisms underlying this decrease in *T* *β* *R*-*I* *e*xpression in HNSCCs, we investigated the possible presence of mutations and aberrant methylation of the *T* *β* *R*-*I* *g*ene promoter.

## 2. Materials and Methods

### 2.1. Patient Population

Fifty Puerto Rican patients who had undergone surgery for HNSCC were included in this study. Institutional Review Board approvals were obtained from both the University of Puerto Rico Medical Sciences Campus and the Moffitt Cancer Center. Complete sociodemographic information was obtained for all patients (Table 1). There were 42 males (84%) and 8 females (16%) ranging in age from 38 to 84 years with a mean of 61.5 years. Clinicopathological data collected included stage, tumor site, degree of tumor differentiation, treatment method, date and site of tumor recurrence and date and cause of death.

### 2.2. Immunohistochemistry of TGF-*β* Receptors

Immunohistochemistry (IHC) protocols have been previously published [34]. Tissue sections, 4 *μ*m in thickness, were deparaffinized, rehydrated, incubated with 0.3% peroxide, washed in water and subjected to antigen retrieval. Blocking serum was applied and the slides blotted. Sections were then incubated overnight, at 4^∘^C in a humidified atmosphere, with a primary anti *T* *β* *R*-*I* antibody (Santa Cruz Biotechnology, Santa Cruz, Calif, USA) at a 1:100 dilution. Sections were then rinsed with PBS and incubated with the secondary antibody for 30 minutes at room temperature. Detection was performed using the Vectastain ABC kit, rabbit IgG, Elite series (Vector Laboratories, Burlingame, Calif, USA). Antibody binding was visualized using 3,3′-diaminobenzidine. Sections were counterstained with hematoxylin. On each run, tissue sections, known to express the protein, were used as positive controls and negative controls were incubated with PBS instead of the primary antibody. Expression of *T* *β* *R*-*I* was evaluated in the tumor and in adjacent nonneoplastic epithelium. Quantitation was performed, following the method recommended by the College of American Pathologists, as follows: 0 = no expression; 1+ = <25% cells; 2+ = 26–50% cells; 3+ = >50% cells.

### 2.3. Genomic DNA and RNA Isolation

Fresh-frozen tissue samples were macrodissected to obtain a 90–95% purity of nonnecrotic tumor and noninvolved adjacent nonneoplastic epithelium. Genomic DNA was isolated from both the tumor and adjacent nonneoplastic tissue using the DNA Isolating Kit for Cells and Tissues (Roche Applied Science, Hague Road, Ind, USA). DNA from peripheral blood lymphocytes was isolated using the QIAmp Blood DNA Maxi Kit from Qiagen Inc. (Valencia, Calif, USA). For semiquantitative RT-PCR analysis, total RNA was isolated from frozen tumor tissue, using the RNeasy Midi Kit (Qiagen) and following manufacturer's specifications.

### 2.4. T*β*R-I Methylation Status

The methylation status of the promoter region of *T* *β* *R*-*I* was assessed by restriction enzyme-mediated PCR (MSRE) and methylation-specific PCR (MSP). Genomic DNA, isolated from peripheral blood lymphocytes (PBL), served as normal control. The DNA from both tumor and nonneoplastic epithelium (150–200 ng) was digested for 6 hours with *Bst*UI (New England Biolabs, Ipswich, Mass, USA) according to conditions specified by the manufacturer. PCR amplification of unmodified DNA and restriction digests were performed in a total volume of 25 *μ*L containing 1U FastStart Taq DNA polymerase using the PCR buffer supplied by the manufacturer (Roche Applied Science, Indianapolis, Ind, USA) with the addition of GC-RICH Resolution Solution as recommended (200 *μ*M dNTP, 200 ng of DNA template, 2 mM MgCl2, and 0.4 *μ*M of each primer). The sequences of sense and antisense primers have been reported previously [32]. Reactions were hot-started at 95°C for 5 minutes. This was followed by 35 cycles of 30 seconds at 95°C, 90 seconds at 55°C, and 90 seconds at 72°C. Amplification products were separated in a 2% agarose gel, stained with ethidium bromide, and documented using the Gel Doc 1000 System with Molecular Analysis Software (BioRad, Hercules, Calif, USA). Amplification products were detected when digestion of the tumor genomic DNA with methylation-sensitive restriction endonuclease *Bst*UI was inhibited by the presence of the methylated CpG motifs. Since incomplete digestion of genomic DNA with *Bst*U1 could result in false positives, the procedure was performed twice to ensure full digestion and reproducibility of results.

Aberrant methylation was independently tested by methylation-specific PCR (MSP). This method relies on the conversion of unmethylated cytosine to uracil by sodium bisulfite. Genomic DNA (1 *μ*g) was modified by bisulfite treatment using the CpGenome DNA modification kit following manufacturer's instructions (Intergen Co., Purchase, NY, USA). The primer sets used anneal specifically to the methylated bisulfite-modified DNA and are described elsewhere [32]. PCR was performed using 5 *μ*L of each bisulfite-modified DNA as template in a 25 *μ*L volume containing 1U FastStart Taq DNA polymerase in the buffer supplied by the manufacturer (Roche Applied Science), with the addition of GC-RICH Resolution Solution as recommended (200 *μ*M dNTP, and 0.4 *μ*M of each primer, 2 mM MgCl_2_ and 5% DMSO). The reaction mixture was incubated at 95°C for 5 minutes and then subjected to 35 cycles of amplification consisting of 1 minute at 95°C, 90 seconds at 55°C, and 90 seconds at 72°C and a final extension of 7 minutes at 72°C. The amplified fragments were subjected to electrophoresis in a 3% agarose gel, stained with ethidium bromide and documented using the Gel Doc 1000 System with Molecular Analysis Software (BioRad). 

### 2.5. T*β*R-I RNA expression

For semiquantitative RT-PCR analysis, total RNA was isolated from macrodissected (enriched) fresh frozen tumor tissue, suitable for mRNA analysis, using the RNeasy Midi Kit (Qiagen, Valencia, Calif, USA) following manufacturer's specifications. Complimentary DNA (cDNA) was prepared from each sample using M-MLV reverse transcriptase (Gibco BRL, Life Technologies, Carlsbad, Calif, USA). Primers for the *T* *β* *R*-I gene were designed using the Primer 3 program at http://frodo.wi.mit.edu/cgi-bin/primer3/primer3_www.cgi and tested for uniqueness in BLAST. cDNA was amplified with the *T* *β* *R*-*I* gene primers 5′-GGTCTTGCCCATCTTCACAT-3′ (sense) and 5′-TTGCTCCAAACCACAGAGTG-3′ (antisense). Primer amplification was performed by adding 2 *μ*L of each cDNA sample to a final reaction mixture of 25 *μ*L containing 1U FastStart Taq DNA polymerase in the buffer supplied by the manufacturer (Roche Applied Science), with the addition of GC-RICH Resolution Solution as recommended (200 *μ*M dNTP, 0.4 *μ*M of each primer, and 2 mM MgCl_2_). PCR cycle conditions were experimentally determined in order to maintain a linear stage. The PCR conditions were: 95°C for 5 minutes followed by 30 cycles of amplification consisting of 1 minute at 95°C, 45 seconds at 59°C, 1 minute at 72°C and a final extension of 7 minutes at 72°C. *ACTB* was used to normalize the amount of mRNA present in each sample. Control reactions without DNA were included in each assay. The PCR products were separated in a 2.5% agarose gel, stained with ethidium bromide, and documented with the Gel Doc 1000 System and Molecular Analysis Software (BioRad). Measurements were performed twice to ensure reproducibility of results. The levels of gene transcripts were quantified as the ratio of intensity of target signal to the intensity of *ACTB* signal, using the Bio-Rad's Quantity One software package.

### 2.6. T*β*R-I Genotyping

Genomic DNA (gDNA) was amplified using primers specific for exons 1 and 7. Exon 1 (>70% GC rich) was amplified using the primers 5′-GAGGCGAGGTTTGCTGGGTGAGGCA-3′; 5′-CATGTTTGAGAAAGAGCAGGAGCGAG-3′, and the Advantage-GC Genomic PCR Kit from Clonetech laboratories (Mountain View, Calif, USA). Exon 7 was amplified using the primers: 5′-AAAGGAGGTTCATCCAAATA-3′; 5′CAACTTCTGATGCTCATGACAAA-3′. PCR products were generated in a volume of 50 *μ*L containing 500 ng of genomic DNA, 10X PCR Buffer (100 mM Tris-HCl [pH 8.3], 500 mM KCl, 15 mM MgCl2, 0.1% Gelatin), 0.25 mM each of dNTP, 100 ng of each primer, 0.056 *μ*M TaqStart Antibody (Clontech Laboratories, Palo Alto, Calif, USA) and 2.5 Units of Taq (Gibco/BRL, Gaithersburg, Md, USA). The PCR parameters were as follows: initial denaturing at 60°C for 3 minutes and 94°C for 5 minutes, followed by 30 cycles of 94°C, 1 minute; 55°C, 1 minute; 72°C, 1 minute, followed by one extension cycle of 94°C, 1 minute; 55°C, 2 minutes; 72°C, 5 minutes. The PCR fragments were purified using the Freeze and Squeeze DNA Purification Kit (BioRad).

### 2.7. DNA Sequencing

DNA sequencing was performed at the University of Pennsylvania DNA Sequencing Facility on an ABI (Applied Biosystems) sequencer 3730XL with BigDye Taq FS Terminator V 3.1.

### 2.8. Statistical Analysis

The Fisher's Exact test was used for correlation analysis. A *P* < .05 was considered statistically significant.

## 3. Results

**Figure 1:**
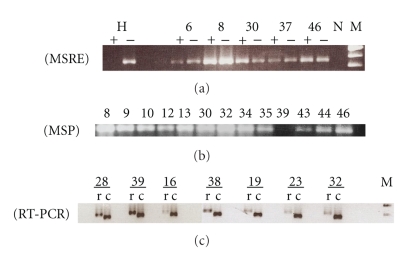
Analysis of *T* *β* *R*-*I* promoter status and gene function in HNSCCs. (a) Representative examples of restriction enzyme-mediated PCR (MSRE) experiments. Analyses were performed for each tumor in the presence (+) and in the absence (−) of *Bst*UI as described in Materials and Methods. Presence of PCR products in (+) lanes indicates methylated DNA. Methylation of *T* *β* *R*-*I* was detected for carcinomas 6, 8, 30, 37, and 46. A positive control of peripheral blood lymphocytes DNA (H) shows unmethylated DNA. A negative (N) control without DNA was used in each assay. M: molecular size marker 100 bp. (b) Methylation-specific PCR for bisulfite-modified DNA that was amplified with primers specific for methylated alleles, as described in Materials and Methods. The presence of PCR products (Lanes 1 to 9 and 11 to 12) is indicative of a methylated *T* *β* *R*-*I* gene promoter. Lane 10 (HNSCC no. 39) shows an unmethylated DNA. (c) Semiquantitative RT-PCR analysis of *T* *β* *R*-*I* gene expression in representative samples of HNSCCs. Expression of *ACTB* gene was used as a control for RNA integrity. Relative mRNA level was normalized based on that of *β*-actin (153 bp). The length of the *T* *β* *R*-*I* PCR product is 186 bp. The agarose gel image was taken from a 30-cycle PCR. *T* *β* *R*-*I* (a) and *ACTB* (b) PCR products were visualized after electrophoresis through 2.5% agarose. HNSCC samples 28, 16, 38, 19, 23, 32 have lost or show reduced mRNA expression. HNSCC sample 39 had preserved mRNA expression. M: molecular size marker 50 bp.

 In order to investigate potential mechanisms of inactivation of the TGF-*β* signaling pathway in squamous cell carcinomas of the head and neck (HNSCC), we examined the methylation and mutation status of the *T* *β* *R*-*I*gene in HNSCC samples from 50 patients. Of these, 42 HNSCCs (84%) were analyzed, by IHC, in archived formalin-fixed, paraffin-embedded tissue sections. Twenty-five (50%) fresh frozen samples, suitable for mRNA analysis, were tested for RNA expression by semiquantitative RT-PCR. The frequency of *T* *β* *R*-*I* promoter aberrant methylation was detected using restriction enzyme-mediated (*Bst*UI) PCR and methylation specific PCR (MSP). The results of all three methods, immunohistochemistry, gene expression, and methylation analyses, are summarized, in the context of clinical-pathological features, in Tables 1 and 2. We observed no statistically significant associations between the results obtained by any of the three methods and patient's sex, tumor anatomical location, degree of differentiation, or tumor stage (Table 1). Representative results of the molecular analysis are shown in Figures 1(a)–1(c). The presence of amplified products in *Bst*UI-digested DNA (lanes with the + sign) indicates that the *T* *β* *R*-*I* promoter is methylated in the tumor (Figure 1(a); samples 6, 8, 30, 37, and 46). Lack of *T* *β* *R*-*I* PCR product in normal lymphocytes, treated with methylation-sensitive *Bst*UI, is indicative of an unmethylated promoter cleaved by the restriction enzyme (Figure 1(a), lane H). In our series, 31 samples (62%) showed aberrant methylation of the *T* *β* *R*-*I* gene promoter. Both methods detected hypermethylation in 30 HNSCCs and only by MSP in one additional sample [Figure 1(b), no. 43]. MSP is the most sensitive of the two methods and can detect one copy of methylated DNA in 1000 (0.1%) unmethylated copies of genomic DNA [35]. The frequency of *T* *β* *R*-*I* hypermethylation was highest in the oropharynx (80%) and lowest in the hypopharynx (50%).

**Figure 2:**
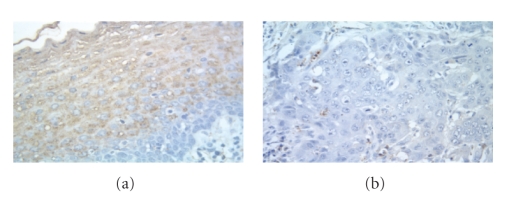
Immunohistochemistry of *T* *β* *R*-*I* protein in HNSCCs (200X). (a) Immunohistochemical detection of *T* *β* *R*-*I* protein (brown signal in nonneoplastic epithelium adjacent to HNSCC). (b) Lack of *T* *β* *R*-*I* protein staining in the HNSCC.

To establish if there was a relationship between methylation and expression of mRNA or protein, we simultaneously analyzed the HNSCCs by RT-PCR (Figure 1(c)) and IHC (Figure 2). Of the 42 tumors tested by IHC, 35 (83%) completely lost protein expression, 5 (12%) showed a reduction of expression compared with adjacent nonneoplastic tissue and in two cases (5%) the tumor showed no reduction of protein expression (Table 2; Figure 2). Of the 7 IHC-positive cases, 3 (49%) showed abnormal methylation and 4 (57%) did not (Table 3). On the other hand, of the 35 IHC-negative tumors, 24 (69%) were aberrantly methylated and 11 (31%) were not. Methylation was in agreement with IHC in 64% of the cases but no strong correlation (*P* = .389) was observed (Table 3). This lack of agreement has been reported in previous studies [36] and thought to be the result of subjective interpretation of IHC results with no uniformly accepted threshold for positivity. With regard to gene expression, of the total of 21 tumors tested, 18 (90%) showed complete loss or downregulation of mRNA expression and 3 (14%) were fully expressed. Of the 18 with altered mRNA expression 17 (94%) lost protein expression and 1 (6%) did not (Table 3). This suggests that decreased protein expression was likely due to downregulation of gene expression. In these 21 cases, an agreement with IHC results was observed on 90% of the cases, and this correlation was statistically significant (*P* = .042).

**Figure 3:**
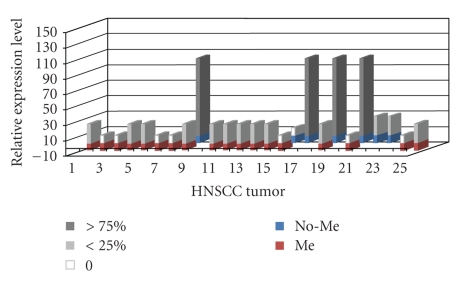
Schematic depiction of *T* *β* *R*-*I* expression by semiquantitative RT-PCR, and hypermethylation by MSP. Tumors with methylated (red bars) and nonmethylated (blue bars) genes are depicted in association with *T* *β* *R*-*I* levels of expression. Columns (white, grey, and dark grey) correspond to relative expression levels (arbitrary units) of *T* *β* *R*-*I* determined by semiquantitative RT-PCR. Methylated genes are associated with lower expression levels of the *T* *β* *R*-*I* gene.

Also, a strong correlation was found between methylation status and *T* *β* *R*-*I* mRNA expression detected by comparative RT-PCR analysis using the ACTB transcript as an internal standard (Figure 1(c)). A 186 bp fragment of the *T* *β* *R*-*I* gene transcript was generated and compared with a 153 bp transcript of the *ACTB* gene. Complete expression of *T* *β* *R*-*I* transcripts was observed in four samples (no. 25, 34, 36, and 39) in concordance with lack of hypermethylation of the gene promoters (Figure 3, lanes 9, 17, 19, and 20). *T* *β* *R*-*I* mRNA expression was reduced or absent in 21 of the 25 tumors tested (84%) and, in these samples, a concordance (*P* = .003) between *T* *β* *R*-*I* gene promoter hypermethylation and *T* *β* *R*-*I* gene expression was observed (Table 4). The loss of mRNA expression in HNSCCs no. 33, 39, and 40 (Figure 3, lanes 16, 22, 23), which lack *T* *β* *R*-*I* aberrant methylation, could be explained by other mechanisms such as epigenetic histone alterations [37].

Of the 25 tumors in which aberrant methylation and gene expression were simultaneously studied (Table 4), 18 (72%) are methylated and 7 (28%) are not methylated. Also, of the 25 tumors, 4 (16%) show normal gene expression, 12 (48%) had partial loss of gene expression, and 9 (36%) show complete loss of gene expression. Of the 18 that are methylated 10 (55%) show downregulation of the gene and 8 (44%) have completely lost gene expression. In the 4 tumors in which the promoter is not methylated the gene is fully expressed indicating that lack of methylation correlates with normal gene expression. Of the 3 remaining tumors in which the promoter was not methylated, 1 (33%) showed no gene expression.

Later, we found that in this case (no 33) the gene has a mutation in exon 1. Another tumor not expressing the gene (no 40) showed a mutation in exon 7. Finally, in another tumor (no 41) the promoter is not methylated and there are no detectable mutations but the gene is downregulated and protein expression is lost. 

Mutations in the *T* *β* *R*-*I* gene have been identified in ovarian, pancreatic, lung, and breast carcinomas [20–25]. Previous studies, however, showed that mutations within the coding sequence of *T* *β* *R*-*I* are rare in HNSCC [26]. We examined twenty-five HNSCC for mutations in the *T* *β* *R*-*I* gene by PCR and direct sequencing of the PCR products. Of the twenty-five samples, 13 (nos. 10, 11, 12, 22, 24, 26, 27, 29, 31, 33, 38, 40, and 50) belonged to this cohort of HNSCC patients and the other 12 samples (data not shown) were from HNSCC patients treated at Moffitt Cancer Center.

We confirmed that mutations of the *T* *β* *R*-*I* gene are, indeed, rare. Sequencing revealed polymorphic sequence changes in only two tumors. Both tumors are from our series (no. 33 and no. 40). An intronic G/A variant, 24 bp downstream of the exon/intron 7 boundary, was detected in sample no. 33. This polymorphism has been associated with various cancer types [20–25]. In addition, a nine-base pair deletion in exon-1, [del(*G* *G* *C*)_3_] , was identified in sample no. 40 in both the tumor and in nontumor genomic DNA. This deletion could represent the germline deletion identified previously by Pasche et al. [38].

## 4. Discussion

 Epigenetic mechanisms (DNA methylation, histone modifications, and chromatin remodeling) are altered in cancer and play a central role in the initiation and progression of the disease [11, 12, 37]. The pattern of aberrant hypermethylation is specific for each tumor type [12]. Our results implicate, for the first time, the *T* *β* *R*-*I*gene as a target for inactivation by aberrant methylation in head and neck squamous cell carcinoma.

Disruption of the TGF-*β* signaling transduction pathway has been shown in a significant subset of human cancers. Key steps are the formation of a heterodimeric complex between receptors type II and type I, phosphorylation of type I receptor and activation of the downstream targets. The fact that aberrant methylation of *T* *β* *R*-*I* is likely to be an important step in cancer progression is supported by a similar observation in gastric cancer cell lines and in primary gastric adenocarcinomas [32, 33]. Our studies confirm previous studies by Pinto et al. [33] who demonstrated that aberrant methylation of the *T* *β* *R*-*I* gene, in gastric tumors, is associated with loss of gene transcription. Gene inactivation resulted on loss of RNA and protein expression (Tables 3 and 4). Our study also reveals a significant association between promoter hypermethylation and loss of gene expression. However a strong association with reduction or loss of protein expression could not be established. Loss of protein expression, measured by IHC, appears not to be a good predictor of DNA methylation-dependent gene silencing [36, 39] suggesting that different gene silencing mechanisms such as histone modifications are likely to occur [40].

Recently, inactivation of *T* *β* *R*-*I* *I* in lung cancer cell lines has been associated with alterations in the chromatin structure of the promoter region, most probably by histone deacetylation [37]. DNA methylation at the *T* *β* *R*-*I* *I* promoter of exon 1 was also detected in a group of cells suggesting that aberrant methylation also played a role in the loss of *T* *β* *R*-*I* *I* expression. It would be of interest to determine whether, in these tumors, alterations of the chromatin structure contribute to the inactivation of *T* *β* *R*-*I*. On the other hand, the aberrant methylation, detected in one sample, only by MSP, can be explained by the inherent sensitivity of the method which can detect methylated alleles in 0.1% of a total DNA sample [35].

Mutations of *T* *β* *R*-*I* have been detected in metastatic HNSCCs [27]. In our series, we found two tumors with mutations in the coding region of *T* *β* *R*-*I*. In HNSCC no. 40, an Int7G24A was detected in exon 7. The Int7G24A variant in *T* *β* *R*-*I* has been detected more frequently in patients with carcinomas of kidney and bladder than in normal age-matched controls [24]. In a study of HNSCCs, 17% of the carcinomas were heterozygous for Int7G24A [26]. This is consistent with our data, since we detected this alteration in only one of the tumors examined. Also, we detected a common polymorphism of *T* *β* *R*-*I*, *T* *G* *F* *β* *R*1*6*A* *,* consisting of a deletion of 3 alanines within a 9-alanine repeat at the 3′ end of the exon 1 coding sequence [38, 41]. Previously, Pasche et al. [38] showed that *T* *G* *F* *β* *R*1*6*A* is somatically altered in cancer and functions as a tumor susceptibility allele. More recently, Pasche et al. [41] reported that the *T* *G* *F* *β* *R*1*6*A* variant is rarely found (1.8%) in primary HNSCC. This alteration, found in one of our samples (no. 33), has been described in many cancer types. A recent meta-analysis of several large cohorts, which included a total of 13 113 individuals [42], supports the hypothesis, proposed by Pasche [38], that *T* *G* *F* *β* *R*1*6*A* is associated with increased cancer risk. More recently, Bian et al. demonstrated that somatic acquisition is a critical event in the early stages of cancer development associated with field cancerization [43].

Our findings show that *T* *β* *R*-*I* is a primary target for aberrant methylation. This can explain previous observations of *T* *β* *R*-*I* loss of expression. Studies by Mi et al. [44] showed that TGF-*β* resistance, at late stages of HPV16-mediated transformation of human keratinocytes, is the result of a loss of expression of *T* *β* *R*-*I*. This significant decrease in mRNA levels can be explained by hypermethylation of the *T* *β* *R*-*I* promoter region. Similarly, Marsit et al. [45] found that promoter methylation in the secreted frizzled-related protein 4 (*SFRP4*) gene was independently associated with the presence of HPV16 viral DNA in HNSCC. SFRPs are antagonists of Wnt signaling that inhibit Wnt receptor binding and downregulate pathway signaling in development. *SFRP4* has been found frequently methylated in colorectal cancer and in chronic lymphocytic leukemia [46, 47]. We have previously shown a high frequency of HPV16 infection in Puerto Ricans with HNSCCs [4]. Studies are under way to ascertain if infection with HPV16 facilitates hypermethylation of genes associated with cancer in HNSCCs.

Analysis of the association between *T* *β* *R*-*I* aberrant methylation and prognostic factors (Table 1) such as age, gender, stage, and tumor site showed no statistically significant correlations. However, *T* *β* *R*-*I* aberrant methylation was shown in early (I and II) and advanced (III and IV) tumor stages suggesting that epigenetic disruption of TGF-*β* signaling by aberrant methylation might contribute to the progression of HNSCCs.

## 5. Conclusion

 Our findings indicate that epigenetic silencing is the main mechanism of inactivation of *T* *β* *R*-*I* in HNSCCs. Gene methylation occurs frequently in human cancers and has been demonstrated early in tumorigenesis. Several studies have shown that promoter methylation of cancer genes is specific to preneoplastic and neoplastic cells [48]. DNA methylation may be present before the cancer is detected by conventional methods and, thus, can simultaneously provide diagnostic and prognostic information. PCR-based detection of hypermethylated genes both in tissue and in body fluids such as urine or blood can be useful in cancer diagnosis. For a biomarker to be useful in the detection of early cancer, however, it has to discriminate between neoplastic and nonneoplastic cells.

More comprehensive studies, including tumors and matched controls, are needed to address the sensitivity, specificity, and predictive value of *T* *β* *R*-*I* methylation-based cancer detection. Nevertheless, *T* *β* *R*-*I* hypermethylation has already shown to have a significant degree of specificity in gastric cancer, and it appears that the same is very likely for head and neck cancer. Different frequencies of a variety of methylated cancer genes are reported in different cancer types suggesting that accurate diagnosis of a specific cancer type may require the detection of a panel of hypermethylated genes present at high frequency in the tumor cells. Furthermore, gene methylation can potentially be evaluated in the patients sera to detect early recurrences in those primary tumors that display a given methylation pattern. Thus, in addition to *CDKN2A*, *T* *β* *R*-*I* gene could be added to the list of cancer genes that must be tested for methylation-based detection of head and neck cancer.

## Figures and Tables

**Table 1:** Relationship between clinicopathological features of HNSCC patients and *T* *β* *R*-*I*gene promoter aberrant methylation.

	No. Patients (%)	Methylation (%)	*P*
All patients	50 (100)	31( 62)	
Male	42 ( 84)	27 (64)	
Female	8 (16)	4 (50)	0.450
Site			
Oral Cavity	22 (44)	13 (59)	
Oropharynx	5 (10)	4 (80)	
Hypopharynx	6 (12)	3 (50)	
Larynx	17 (34)	11 (65)	0.756
Tumor differentiation			
Well differentiated	13 (26)	10 (76)	
Mod. differentiated	30 (60)	19 (63)	
Poorly differentiated	5 (19)	4 (80)	0.649
n.a.	2 ( 4)		
Disease stage			
I	4 ( 8)	3 (75)	
II	7 (14)	5 (71)	
III	13 (26)	8 (62)	
IV	26 (52)	15 (57)	0.917
Median age (range), y	61.54 (38–84)		

Abbreviation: na Not available.

**Table 2:** Correlation between, tumor characteristics, protein and mRNA expression, and promoter methylation. Grading and tumor, lymph node, metastasis, and staging (TNM) are according to the 2002 UICC classification.

Tumor	Age	Site	Differentiation	Stage	Protein expression		∆ Expression	*T* *β* *R*-*I* Methylation	*T* *β* *R*-*I*expression
					Normal	Carcinoma			
1	82	HP	MD	IV	2+	1+	Decreased	NM	
2	67	L	MD	IV	2+	0	Lost	NM	
3	57	L	MD	IV	1+	0	Lost	M	
4	64	L	PD	III	1+	0	Lost	NM	
5	75	L	MD	II	1+	0	Lost	M	
6	79	OC	MD	III	1+	0	Lost	NM	
7	56	OP	MD	IV	2+	1+	Decreased	NM	
8	53	OP	MD	IV	1+	0	Lost	M	
9	71	OC	WD	III	1+	0	Lost	M	
10	84	OP	MD	III	2+	0	Lost	M	2
11	73	L	MD	IV	1+	0	Lost	M	3
12	67	OC	WD	III	2+	0	Lost	M	3
13	38	L	MD	I	1+	0	Lost	M	2
14	48	HP	MD	1V	na	na	na	NM	
15	62	OP	MD	II	2+	1+	Decreased	M	
16	58	L	MD	III	3+	0	Lost	M	2
17	66	OC	PD	III	1+	0	Lost	NM	
18	50	L	SCC	IV	2+	0	Lost	NM	
19	55	OC	MD	IV	na	na	na	M	3
20	58	OC	WD	IV	2+	0	Lost	NM	
21	46	OC	WD	III	3+	0	Lost	M	
22	66	OC	MD	I	1+	0	Lost	M	3
23	70	L	WD	IV	2+	0	Lost	M	2
24	51	OP	WD	IV	1+	0	Lost	M	
25	74	HP	MD	IV	na	na	na	NM	1
26	56	OC	MD	I	3+	0	Lost	M	2
27	66	HP	PD	III	1+	0	Lost	M	2
28	68	OC	MD	II	2+	0	Lost	M	2
29	81	OC	MD	IV	3+	0	Lost	M	3
30	55	L	MD	II	2+	0	Lost	M	
31	44	L	MD	IV	1+	0	Lost	M	2
32	55	OC	MD	IV	1+	0	Lost	M	3
33	84	OC	SCC	I	na	na	na	NM	3
34	56	OC	WD	II	1+	1+	Unchanged	NM	1
35	74	HP	PD	IV	1+	0	Lost	M	2
36	60	OC	PD	II	3+	0	Lost	NM	1
37	60	L	WD	IV	3+	0	Lost	M	
38	56	L	WD	II	na	na	na	M	3
39	56	L	MD	IV	3+	1+	Decreased	NM	1
40	48	L	MD	III	2+	0	Lost	NM	2
41	62	OC	MD	IV	1+	0	Lost	NM	2
42	65	L	WD	IV	3+	0	Lost	NM	
43	47	OC	MD	III	3+	1+	Decreased	M	3
44	75	L	MD	III	na	na	na	M	
45	51	OC	WD	IV	3+	3+	Unchanged	M	
46	49	OC	WD	IV	na	na	na	M	
47	70	OC	MD	IV	na	na	na	NM	
48	47	OC	MD	III	1+	0	Lost	NM	
49	50	HP	MD	IV	1+	0	Lost	M	2
50	72	OC	WD	IV	2+	0	Lost	M	

OC = Oral cavity, OP = Oropharynx, HP = Hypopharynx, LA = Larynx, na = Not available. 1 = Fully expressed; 2 = Down-regulated; 3 = Not expressed. WD = Well differentiated, MD = Moderately differentiated, PD = Poorly differentiated, SCC = Squamous cell carcinoma

**Table 3:** Relationship between *T* *β* *R*-*I* gene promoter aberrant methylation and *T* *β* *R*-*I* protein expression.

	Methylation		Gene expression		
Protein expression (IHC)	+	−	Complete	Partial	None
Positive	3	4	2	0	1
Negative	24	11	1	12	5
Total	27 (64%)	15 (36%)	3 (14%)	12 (57%)	6 (29%)
*P* value	0.389		0.042		

**Table 4:** Relationship between *T* *β* *R*-*I* gene promoter aberrant methylation and gene expression.

	Gene expression			Total (%)
	Complete	Partial	None	
Methylated	0	10	8	18 (72%)
Not Methylated	4	2	1	7 (28%)
Total (%)	4 (16%)	12 (48%)	9 (36%)	25 (100%)
